# Artificial Intelligence-Induced Deskilling in Interventional Pulmonology: An International Cross-Sectional Survey on Risk Perception and Mitigation Strategies

**DOI:** 10.3390/arm94040048

**Published:** 2026-07-20

**Authors:** Guido Marchi, Lorenzo Corbetta

**Affiliations:** 1 Pulmonology Unit, Cardiothoracic and Vascular Department, University Hospital of Pisa, Via Paradisa 2, 56124 Pisa, Italy; 2 Department of Experimental and Clinical Medicine, University of Florence, Viale Morgagni 63, 50134 Florence, Italy

**Keywords:** artificial intelligence, interventional pulmonology, bronchoscopy, deskilling, automation bias, upskilling inhibition, simulation-based training, medical education, procedural competence

## Abstract

**Highlights:**

**What are the main findings?**

- Interventional pulmonologists internationally perceive AI-induced deskilling as a relevant and emerging risk, with 73% concerned about procedural skill erosion and 83% about upskilling inhibition in specialist training, even prior to widespread clinical AI deployment.
- A 43-percentage-point gap between prior familiarity with automation bias (38%) and its recognized clinical relevance after definition provision (81%) reveals a substantial conceptual literacy deficit among IP practitioners.

**What are the implications of the main findings?**

- Training programmes and continuing professional development pathways in interventional pulmonology should incorporate structured modules on automation bias, cognitive offloading, and safe human-AI interaction principles.
- Scientific societies and credentialing bodies should proactively develop governance frameworks—including AI-free training mandates, simulation-based competency maintenance, and minimum non-AI-assisted procedural volume requirements—in anticipation of the expected progressive integration of AI applications into clinical practice.

**Abstract:**

Artificial intelligence (AI) is progressively reshaping interventional pulmonology (IP), yet its potential to erode procedural and cognitive competencies through AI-induced deskilling remains poorly characterized in this specialty. An international, observational, cross-sectional survey was conducted in May 2026 among 118 expert interventional pulmonologists from 10 different countries across 5 continents. Participants completed a structured questionnaire comprising five demographic items and 12 Likert-scale statements addressing deskilling risk perception and mitigation attitudes; percentage agreement was calculated for each item (scores 4–5). High perceived clinical value of AI was reported (87%), alongside substantial concern for procedural deskilling (73%) and upskilling inhibition (83%). Familiarity with automation bias was limited (38%), yet its clinical relevance was widely recognized after definition provision (81%)—a gap of 43 percentage points. Strong support emerged for AI-free training (84%), simulation-based training (86%), and longitudinal performance monitoring (78%). Concern for institutional fragility in the absence of AI was expressed by 74%, and governance frameworks, including minimum non-AI-assisted procedural volume requirements, were endorsed by 70%. Deskilling was identified as a high research priority by 89%. These findings indicate that AI-induced deskilling is perceived as a relevant and emerging risk by expert interventional pulmonologists internationally, even before the widespread clinical deployment of AI technologies. Although the extent to which these concerns will translate into measurable effects on procedural competence is currently uncertain, the results underscore the need for prospective research, educational initiatives, and appropriate governance frameworks to ensure the preservation of core procedural skills.

## 1. Introduction

Artificial intelligence (AI) is rapidly transforming the practice of interventional pulmonology (IP), with emerging applications spanning AI-assisted lung nodule detection and radiomics-based risk stratification, robotic and computer-aided bronchoscopic navigation, AI-enhanced endobronchial ultrasound (EBUS) image interpretation, machine learning–supported rapid on-site evaluation and cytopathology assessment, and decision-support tools for ultrasound-guided pleural procedures [1,2,3,4,5,6,7,8,9,10,11,12,13,14,15,16,17,18]. The integration of these technologies into procedural workflows is widely anticipated to enhance diagnostic accuracy and broaden procedural access in the coming years. As this trajectory accelerates, a critical and underappreciated risk warrants systematic attention: the possibility that sustained AI exposure may progressively erode the procedural, perceptual, and cognitive competencies that define expert-level interventional practice.

The phenomenon of AI-induced deskilling—defined as the degradation of previously acquired clinical competencies due to reduced independent practice consequent to automation—has been systematically examined in the recent literature [18,19,20]. Three interdependent mechanisms have been identified: automation bias (the tendency to over-trust algorithmic outputs and accept recommendations without adequate critical evaluation), cognitive offloading (the progressive delegation of perceptual and reasoning tasks to the machine, leading to atrophy of independent analytical processes), and upskilling inhibition (the reduction in opportunities for trainees to develop expert-level skills through autonomous experience) [21]. Beyond individual cognitive effects, deskilling also carries organizational and systemic dimensions, threatening team situational awareness, the mentorship pipeline, and institutional resilience when AI systems are unavailable or produce erroneous outputs [18,22].

The most instructive empirical evidence currently available comes from an adjacent procedural specialty. A multicenter observational study by Budzyń and colleagues demonstrated that sustained exposure to AI-assisted colonoscopy was independently associated with a significant decline in adenoma detection rate during subsequent non-AI-assisted procedures—from 28.4% before AI implementation to 22.4% after (OR 0.69, 95% CI 0.53–0.89; *p* = 0.0089)—while AI-assisted performance remained stable at 25.3% [23]. This finding—the first prospective empirical demonstration of procedural deskilling attributable to AI exposure in a clinical setting—has attracted regulatory and bioethical attention and serves as a critical proof-of-concept for procedural specialties more broadly [24].

Structural features of IP render the specialty potentially even more susceptible to analogous dynamics: IP procedures depend on continuous real-time integration of perceptual cues, spatial reasoning, and contextual clinical judgement—precisely the cognitive and psychomotor domains most susceptible to atrophy under conditions of habitual AI reliance. Many IP procedures are performed at moderate volumes even in specialized centers; learning curves are steep and protracted, and navigational AI systems directly mediate the perceptual-motor feedback loops on which skill consolidation depends. These features differentiate IP from specialties in which AI primarily assists retrospective or asynchronous interpretation, such as radiology or histopathology, where the clinician’s perceptual engagement with the primary data is not displaced in real time. In IP, by contrast, navigational and image-interpretation AI tools are interposed directly within the perception-action loop during the procedure itself, so that reliance on automated guidance may substitute for, rather than merely supplement, the operator’s own perceptual and psychomotor engagement. Because procedural competence in IP is built through repeated, effortful integration of these perceptual and motor elements, automation bias and cognitive offloading may translate more directly into measurable skill attrition than in specialties where AI outputs are reviewed asynchronously and the clinician retains full independent control of the primary task.

Despite these structural vulnerabilities, no study has yet systematically characterized the perceptions of IP specialists regarding AI-induced deskilling risk at an international level.

This survey aimed to explore such perceptions, assess awareness of key deskilling mechanisms, and evaluate attitudes towards educational and governance mitigation strategies among expert IP practitioners across 10 countries.

## 2. Materials and Methods

### 2.1. Study Design and Population

An international, observational, cross-sectional, questionnaire-based study was conducted in May 2026. The target population comprised interventional pulmonologists from 10 different countries: Australia, Canada, France, India, Italy, Morocco, Spain, Turkey, the United Kingdom, and the United States. Eligible participants were practicing specialists with documented experience in IP procedures, including flexible bronchoscopy, EBUS, robotic bronchoscopy, thoracoscopy, and/or ultrasound-guided pleural interventions. A convenience sample was targeted to ensure broad international representativeness. Respondents were recruited through professional networks affiliated with national and international respiratory societies. Recruitment was based on direct invitations sent to 150 eligible participants identified a priori according to their expertise in IP and related fields. A pragmatic, non-probabilistic target of approximately 10–12 respondents per country was set a priori to achieve balanced international representation across the ten participating countries. No formal a priori sample size calculation was performed, consistent with the descriptive, exploratory, hypothesis-generating design of the study. Participation was entirely voluntary and anonymous, with no collection of personally identifiable or patient-related data. All responses reflected professional opinions on educational content and were recorded in a manner that precluded participant identification.

### 2.2. Survey Instrument

The questionnaire was developed through a structured, iterative process grounded in established frameworks on automation-induced deskilling and cognitive bias, including the deskilling typology proposed by Natali et al. [4], the scoping review by Heudel et al. [5], and empirical evidence from colonoscopy and other medical domains [6]. Items were generated to cover key conceptual domains identified in the literature and subsequently refined to ensure clarity, relevance, and consistency with IP practice. The draft instrument underwent internal review by the study authors, with iterative revisions to improve content validity, reduce ambiguity, and standardize terminology across procedural contexts.

This internal review constituted a face-validity assessment; no cognitive interviewing with prospective respondents external to the authorship team was performed prior to distribution. The final version was structured into coherent thematic sections addressing perceived impacts of AI and automation on clinical decision-making, procedural performance, and training-related aspects of interventional pulmonary practice.

The questionnaire consisted of two sections.

Section 1 collected five demographic and professional variables, including country of practice, gender, age group (<35, 35–44, 45–54, and ≥55 years), years of experience in interventional pulmonology (<5, 5–10, 11–20, and >20 years), and primary clinical setting (academic/university hospital, community hospital, private practice, and mixed practice).

Section 2 included 12 statements rated on a five-point Likert scale (1 = strongly disagree, 2 = disagree, 3 = neither agree nor disagree, 4 = agree, and 5 = strongly agree). The 12 items addressed the following domains: perceived clinical potential of AI in IP (Q1); concern for procedural deskilling consequent to routine AI adoption (Q2), which assessed respondents’ concern that routine AI adoption would reduce previously acquired procedural competence; prior familiarity with the concept of automation bias (Q3); perceived clinical relevance of automation bias after definition provision (Q4); upskilling inhibition in specialist training (Q5), which specifically assessed concern that AI assistance would reduce trainees’ opportunities to develop independent expert-level skills through autonomous practice; perceived need for structured AI-free training sessions (Q6); perceived importance of simulation-based training for competence maintenance (Q7); perceived importance of longitudinal performance monitoring following AI introduction (Q8); perceived inadequacy of current training programmes for safe AI integration (Q9); perceived priority of deskilling as a scientific research agenda item (Q10); perceived risk of institutional fragility and systemic resilience reduction in the event of AI unavailability or malfunction (Q11), grounded in the concept of system embrittlement; and support for formal institutional governance frameworks including minimum non-AI-assisted procedure volume requirements (Q12), informed by analogous aviation regulatory precedents [25].

A summary of the key points is presented in Table 1.

The full detailed questionnaire is available as Supplementary Material (Table S1).

### 2.3. Statistical Analysis

Statistical analysis was performed using descriptive methods. Categorical variables, including demographic and professional characteristics, were summarized as absolute frequencies and percentages. For each Likert-scale item, the level of agreement was operationally defined as the proportion of respondents selecting scores 4 (agree) or 5 (strongly agree), calculated over the total number of valid responses (*n* = 118). The questionnaire enforced a mandatory response to every item prior to submission, precluding item-level missing data; the analytic denominator was therefore constant at *n* = 118 for all 12 items. No inferential statistical testing was conducted, in accordance with the exploratory and hypothesis-generating nature of the survey design. Consequently, all reported percentages should be interpreted descriptively; no statistical comparisons between items, subgroups, or countries were performed, and no claims of statistical significance are made anywhere in this manuscript. All percentages were derived from exact proportions of the full sample and subsequently rounded to the nearest integer for presentation purposes. The complete frequency distribution (absolute number and percentage) across all five response categories for each of the 12 items is reported in the Supplementary Material (Table S1).

## 3. Results

### 3.1. Demographic Characteristics

A total of 118 IP specialists from ten countries participated in the survey, corresponding to a response rate of 78.7% (150 eligible participants invited). Eight countries contributed 12 respondents each (Australia, Canada, France, India, Italy, Turkey, the United Kingdom, and the United States), while two countries contributed 11 respondents each (Morocco and Spain).

The sample comprised 76 male (64.4%) and 42 female (35.6%) respondents. The most represented age group was 35–44 years (*n* = 45; 38.1%), followed by 45–54 years (*n* = 33; 28.0%), ≥55 years (*n* = 21; 17.8%), and <35 years (*n* = 19; 16.1%); the 35–54-year age range collectively accounted for 66.1% of respondents (*n* = 78).

With respect to years of experience in IP, the most prevalent category was 5–10 years (*n* = 39; 33.1%), followed by 11–20 years (*n* = 36; 30.5%), >20 years (*n* = 23; 19.5%), and <5 years (*n* = 20; 16.9%); 83.1% of respondents (*n* = 98) reported five or more years of IP experience.

With regard to primary clinical setting, 56.8% (*n* = 67) worked in academic or university hospitals, 22.0% (*n* = 26) in community hospitals, 11.0% (*n* = 13) in mixed settings, and 10.2% (*n* = 12) in private practice.

A summary of the demographic and professional characteristics of respondents is presented in Table 2.

### 3.2. Survey Item Results

The perceived clinical potential of AI in IP was reported by 87% of respondents (Q1; *n* = 103), indicating a high level of agreement regarding its anticipated clinical utility within the specialty.

Concerns regarding potential procedural deskilling associated with routine AI adoption were reported by 73% of respondents (Q2; *n* = 86), reflecting a substantial proportion of participants expressing concern about this item.

Familiarity with the concept of automation bias prior to receiving its definition was reported by 38% of respondents (Q3; *n* = 45). Following the provision of a standardized definition within the questionnaire, 81% of respondents (Q4; *n* = 96) indicated that automation bias was clinically relevant.

Perceived upskilling inhibition in specialist training was reported by 83% of respondents (Q5; *n* = 98). The perceived need for structured AI-free training sessions was reported by 84% (Q6; *n* = 99), while 86% (Q7; *n* = 101) indicated that simulation-based training is important for competence maintenance.

The importance of longitudinal performance monitoring following AI introduction was reported by 78% of respondents (Q8; *n* = 92). Perceived inadequacy of current training programmes for safe AI integration was reported by 77% (Q9; *n* = 91).

Deskilling was identified as a priority research area by 89% of respondents (Q10; *n* = 105), representing the highest proportion of agreement among all survey items.

Perceived risk of institutional fragility and reduced resilience in the event of AI unavailability or malfunction was reported by 74% of respondents (Q11; *n* = 87). This item addressed perceptions related to system-level vulnerability under conditions of technological dependency.

Support for formal governance frameworks, including minimum non-AI-assisted procedural volume requirements, was reported by 70% of respondents (Q12; *n* = 83), indicating a majority endorsement of structured regulatory approaches to competency maintenance.

Complete results for all 12 survey items are presented in Table 3.

## 4. Discussion

This international survey provides the first exploratory characterization of AI-induced deskilling perceptions among IP specialists across 10 different countries. It should be emphasized at the outset that this survey captures perceptions, concerns, and attitudes rather than objectively measured procedural performance; no procedural competence data were collected, and the findings should not be interpreted as evidence that AI-induced deskilling has been empirically demonstrated in IP. The discussion that follows accordingly refers to perceived risk unless explicitly stated otherwise.

The findings reveal a coherent and internally consistent pattern: high recognition of AI’s clinical potential coexists with substantial concern for procedural skill erosion, broad endorsement of mitigation strategies, and near-universal consensus that deskilling constitutes a high-priority research agenda item for the specialty.

Agreement rates across items ranged from 38% to 89%, indicating variability across conceptual familiarity, risk perception, and governance-oriented constructs, while maintaining overall internal coherence of responses.

These results are particularly noteworthy given that AI adoption in IP remains limited or nascent in most participating contexts, suggesting that awareness of deskilling risk precedes—rather than follows—clinical AI deployment, and that the IP community is cognitively prepared to engage proactively with this challenge.

The most striking finding was the 43-percentage-point discrepancy between prior familiarity with automation bias as a theoretical construct (38%) and recognition of its clinical relevance once the concept was defined within the questionnaire (81%). This pattern closely mirrors findings from qualitative and survey-based studies in other clinical domains [18,23] and suggests that the educational challenge in IP is one of conceptual literacy rather than motivational resistance.

Practitioners appear intuitively sensitive to the operational risk—as confirmed by the high agreement on Q4 after definition—yet lack the formal vocabulary to identify, discuss, and monitor it proactively in their clinical practice. This may have potential implications for curriculum design, suggesting that residency programmes and continuing professional development pathways in IP could consider incorporating structured modules on automation bias, cognitive offloading, upskilling inhibition, and safe human–AI interaction principles.

The highest level of agreement observed in the survey was for deskilling as a priority research area (Q10; 89%), reinforcing the centrality of this theme within respondents’ overall perception framework.

The 83% agreement rate for upskilling inhibition (Q5) also deserves particular attention. IP procedural competence is acquired through a graduated apprenticeship model in which trainees consolidate technical and perceptual skills through progressively autonomous case exposure and deliberate practice. When AI systems provide real-time navigational guidance or automated image interpretation—as increasingly occurs with navigational bronchoscopy platforms and EBUS AI tools—trainees may achieve satisfactory procedural outcomes without ever developing independent perceptual benchmarks or cognitive schema necessary for autonomous performance. This concern mirrors the training ecology effects described in the deskilling literature, whereby AI triage and workflow redesign reduce exposure to the foundational cases essential for skill consolidation [18]. The colonoscopy precedent is instructive: Budzyń et al. demonstrated measurable performance decline in experienced endoscopists—not merely trainees—after sustained AI exposure when subsequently operating without AI assistance [23]. The implication for IP is that perceived deskilling risk may extend across the full spectrum of expertise, although this remains to be empirically confirmed in procedural outcome studies analogous to Budzyń et al.’s colonoscopy investigation.

The consistent endorsement of mitigation-oriented items—AI-free training (84%), simulation-based training (86%), longitudinal performance monitoring (78%), and institutional governance frameworks (70%)—reflects a practitioner community already cognitively prepared to accept structural changes to training and governance.

**Figure 1:**
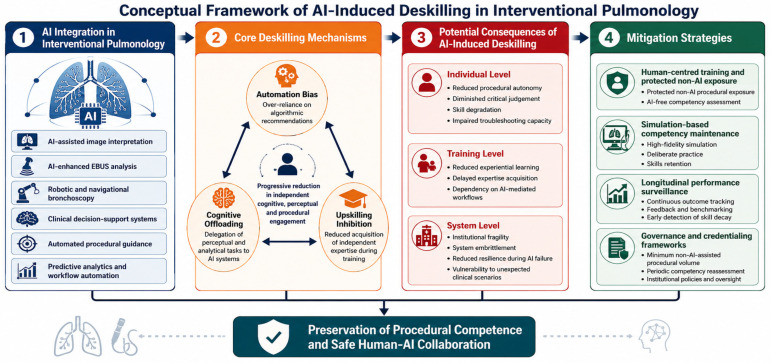
Conceptual framework of artificial intelligence-induced deskilling in interventional pulmonology: mechanisms, consequences, and mitigation strategies. The figure illustrates a four-domain sequential pathway linking AI integration in interventional pulmonology to the preservation of procedural competence and safe human-AI collaboration. (1) AI Integration in Interventional Pulmonology: current and emerging AI-based technologies progressively embedded in procedural workflows, encompassing AI-assisted image interpretation, AI-enhanced endobronchial ultrasound analysis, robotic and navigational bronchoscopy, clinical decision-support systems, automated procedural guidance, and predictive analytics with workflow automation. (2) Core Deskilling Mechanisms: three interacting and mutually reinforcing mechanisms drive a progressive reduction in independent cognitive, perceptual, and procedural engagement—automation bias (over-reliance on algorithmic recommendations without adequate critical appraisal), cognitive offloading (delegation of perceptual and analytical tasks to AI systems), and upskilling inhibition (reduced acquisition of independent expertise during training). (3) Potential Consequences of AI-Induced Deskilling: adverse effects manifest across three hierarchical levels—the individual level (reduced procedural autonomy, diminished critical judgment, skill degradation, and impaired troubleshooting capacity), the training level (reduced experiential learning, delayed expertise acquisition, and dependency on AI-mediated workflows), and the system level (institutional fragility, system embrittlement, reduced resilience during AI failure, and vulnerability to unexpected clinical scenarios). (4) Mitigation Strategies: four interconnected countermeasures are proposed—human-centred training with protected non-AI exposure (including AI-free procedural sessions and AI-free competency assessment), simulation-based competency maintenance (through high-fidelity simulation, deliberate practice, and skills retention protocols), longitudinal performance surveillance (continuous outcome tracking, feedback and benchmarking, and early detection of skill decay), and governance and credentialing frameworks (minimum non-AI-assisted procedural volume requirements, periodic competency reassessment, and institutional policies and oversight). The convergent implementation of these strategies is directed toward the overarching goal of preserving procedural competence and enabling safe human-AI collaboration. AI = artificial intelligence; EBUS = endobronchial ultrasound; IP = interventional pulmonology.

The conceptual interplay between AI adoption in IP, the core deskilling mechanisms identified, their potential consequences at individual, training, and system levels, and the corresponding mitigation strategies endorsed by respondents in this survey is schematically represented in Figure 1.

Simulation-based training and competence assessment have been validated across surgical and interventional specialties as robust strategies for maintaining procedural proficiency, particularly when live case volume is limited. This approach is directly applicable to IP, where bronchoscopy simulators and procedural phantoms are increasingly available and support structured skills acquisition and maintenance.

The endorsement of governance frameworks, including minimum non-AI-assisted procedure volume requirements (Q12; 70%), echoes the aviation regulatory precedent, where the Federal Aviation Administration (FAA) mandates minimum manual flying hours to counteract automation-induced skill fade [25]. Translating this precedent to IP highlights the importance of evidence-based competency thresholds and the availability of validated performance assessment instruments, representing an area of ongoing relevance for respiratory scientific societies and credentialing bodies [26,27,28]. This analogy should be interpreted only as a conceptual reference. While aviation regulation relies on standardized flight-hour metrics and objective, continuously recorded performance indicators, IP lacks universally agreed-upon or consistently implemented definitions of minimum procedural volumes or validated procedure-specific competency thresholds across settings. The FAA framework is therefore intended as an illustrative example of structured governance rather than a directly transferable regulatory model.

The 74% agreement on institutional fragility (Q11) extends the deskilling discourse beyond the individual clinician to the organizational and systemic level. This dimension—aligned with the system embrittlement construct—refers to the progressive institutional loss of capacity to function safely when automated systems are degraded or unavailable. In IP, this concern carries notable clinical relevance: a bronchoscopist whose perceptual and navigational skills have atrophied through habitual AI reliance is precisely the least equipped to manage the unanticipated anatomical variants, equipment failures, or procedural complications that arise in real-world practice [29,30]. The recognition of this risk by nearly three-quarters of respondents suggests that institutional resilience planning should be incorporated into AI governance frameworks from the outset of technology adoption, rather than retrospectively.

Several limitations of this study must be acknowledged. The exploratory design, the convenience sampling methodology, and the variable country-level sample sizes (ranging from 11 to 12 respondents per country) limit the representativeness and generalizability of findings. The cross-sectional design precludes causal inference, and the hypothetical framing of several survey items—anchored in anticipated rather than currently experienced AI integration—introduces potential for speculative or socially desirable responding. The instrument has not undergone formal psychometric validation: no pilot cognitive interviewing, quantitative content validity indexing, or reliability analysis was performed prior to distribution. Content relevance was supported by grounding in established deskilling frameworks and iterative internal review by the study authors, but this process does not substitute for formal instrument validation, and this limitation should be considered when interpreting item-level findings. Additionally, the high proportion of academic-setting respondents (56.8%) may have introduced selection bias, as academic practitioners may be more exposed to AI research and therefore more sensitized to deskilling concerns than community-based peers. Notwithstanding these limitations, the geographic breadth of the sample (10 countries across five continents) and the internally consistent pattern of responses across all 12 survey items support the robustness of the observed signal and warrant confirmation in larger, prospectively designed studies with validated instruments.

## 5. Conclusions

This international exploratory survey indicates that AI-related deskilling is perceived as a relevant and emerging concern among expert interventional pulmonologists across diverse national and institutional settings, even in contexts where clinical AI implementation remains limited.

The observed discrepancy between limited prior familiarity with the concept of automation bias and its substantially higher perceived clinical relevance following definition suggests a potential gap in conceptual exposure within current educational frameworks, highlighting the relevance of structured training on cognitive and human-AI interaction biases within IP curricula.

The consistent endorsement of mitigation-oriented strategies—including AI-free training, simulation-based competence maintenance, longitudinal performance monitoring, and governance frameworks—reflects a broad convergence on potential organizational and educational responses to anticipated changes in clinical practice.

Collectively, these findings support the view that IP is entering a transitional phase in which anticipatory governance, structured education, and competency preservation strategies may play a central role in guiding responsible AI integration.

## Figures and Tables

**Table 1:** Structure and thematic domains of the survey. The questionnaire comprised 12 Likert-scale items (1 = strongly disagree, 2 = disagree, 3 = neither agree nor disagree, 4 = agree, and 5 = strongly agree), exploring interventional pulmonologists’ perceptions of artificial intelligence integration, automation bias, training implications, system resilience, and governance frameworks in interventional pulmonology practice.

Item	Domain	Description
Q1	Perceived clinical potential of AI in IP	Perceived added value of AI in interventional pulmonology practice
Q2	Procedural deskilling risk	Concern regarding loss of procedural skills associated with routine AI use
Q3	Automation bias familiarity	Prior awareness of the concept of automation bias
Q4	Clinical relevance of automation bias	Perceived relevance of automation bias after standardized definition
Q5	Upskilling inhibition	Perceived impact of AI on inhibition of skill acquisition during training
Q6	AI-free training need	Perceived need for structured training sessions without AI assistance
Q7	Simulation-based training	Perceived importance of simulation for competence maintenance
Q8	Longitudinal monitoring	Importance of post-AI implementation performance monitoring
Q9	Training adequacy	Perceived adequacy of current training programmes for AI integration
Q10	Research priority (deskilling)	Priority assigned to deskilling as a research agenda topic
Q11	System resilience risk	Perceived risk of reduced systemic resilience in case of AI unavailability (system embrittlement)
Q12	Governance frameworks	Support for formal governance policies, including minimum non-AI-assisted procedural volume requirements

**Table 2:** Demographic and professional characteristics of respondents. Demographic and professional characteristics of interventional pulmonologists participating in the international survey (*n* = 118), including gender, age distribution, years of experience in interventional pulmonology, and primary clinical setting. Data are presented as absolute numbers and percentages.

Variable	Category	*n* (%)
Gender	Male	76 (64.4)
	Female	42 (35.6)
Age group (years)	<35	19 (16.1)
	35–44	45 (38.1)
	45–54	33 (28.0)
	≥55	21 (17.8)
Years in IP practice	<5	20 (16.9)
	5–10	39 (33.1)
	11–20	36 (30.5)
	>20	23 (19.5)
Clinical setting	Academic/university hospital	67 (56.8)
	Community hospital	26 (22.0)
	Private practice	12 (10.2)
	Mixed practice	13 (11.0)

**Table 3:** Survey responses on AI-induced deskilling perceptions in interventional pulmonology. Responses to 12 Likert-scale items assessing perceptions of artificial intelligence-induced deskilling, automation bias, training implications, system resilience, and governance frameworks among interventional pulmonologists. Agreement was defined as scores of 4–5 on a five-point Likert scale. Data are presented as percentage agreement and absolute number of respondents (*n* = 118). AI = artificial intelligence; IP = interventional pulmonology.

Item	Domain	Agreement, % (Scores 4–5)	*n*
Q1	Perceived clinical value of AI in IP	87	103
Q2	Risk of procedural deskilling with routine AI adoption	73	86
Q3	Prior familiarity with automation bias	38	45
Q4	Clinical relevance of automation bias (post-definition)	81	96
Q5	Upskilling inhibition due to AI over-reliance	83	98
Q6	Need for AI-free training sessions	84	99
Q7	Importance of simulation-based training	86	101
Q8	Importance of longitudinal monitoring	78	92
Q9	Inadequacy of current training programmes for AI integration	77	91
Q10	Deskilling as a research priority	89	105
Q11	Institutional fragility/system resilience risk	74	87
Q12	Support for governance frameworks (min procedural volume)	70	83

## Data Availability

The survey questionnaire used in this study is provided as Supplementary Materials.

## Supplementary Materials

The following supporting information can be downloaded at: https://www.mdpi.com/article/10.3390/arm94040048/s1, Table S1: Full distribution of Likert-scale responses (1 = strongly disagree to 5 = strongly agree) for each of the 12 survey items on AI-related deskilling in interventional pulmonology (*n* = 118 respondents). File S1: Artificial Intelligence-Induced Deskilling in Interventional Pulmonology: An International Cross-Sectional Survey on Risk Perception and Mitigation Strategies.

## Abbreviations

The following abbreviations are used in this manuscript:

AI	Artificial Intelligence
EBUS	Endobronchial Ultrasound
IP	Interventional Pulmonology
OR	Odds Ratio
CI	Confidence Interval
FAA	Federal Aviation Administration
Q	Questionnaire item
