# Supplementary material for: Artificial Intelligence-Induced Deskilling in Interventional Pulmonology: An International Cross-Sectional Survey on Risk Perception and Mitigation Strategies
Source: Adv Respir Med. 2026 Jul 20;94(4):48. doi: 10.3390/arm94040048 (PMC13398125; doi:10.3390/arm94040048)
Supplement: Supplementary file 1 [file arm-94-00048-s001.zip › Survey De-Skilling.pdf]

Artificial Intelligence-Induced Deskilling in Interventional Pulmonology: An International Cross-Sectional Survey on Risk Perception and Mitigation Strategies

- This survey explores clinicians' perceptions of the risk of deskilling (defined as a reduction in procedural competence and independent clinical decision-making associated with reliance on AI-assisted systems during interventional procedures) in the context of increasing integration of AI in interventional pulmonology, together with potential strategies to mitigate this risk.

- The survey is anonymous, voluntary, and no personally identifiable data will be collected. Responses will be used exclusively for research purposes.

- There are no right or wrong answers. Please respond based on your personal experience and opinion.

- Estimated completion time: 3–5 minutes.

Section S1 - Background Information

Q0a. Country of practice

- Australia
- Canada
- France
- India
- Italy
- Morocco

- Spain
- Turkey
- United Kingdom
- United States

Q0b. Gender

- Male
- Female
- Prefer not to say

Q0c. Age group

- <35 years
- 35–44 years
- 45–54 years
- ≥55 years

Q0d. Years of experience in interventional pulmonology

- <5 years
- 5–10 years
- 11–20 years
- 20 years

Q0e. Primary clinical setting

- Academic/University Hospital
- Community Hospital
- Private Practice
- Mixed

Section S2 – Survey Statements

Statements (please indicate your agreement):

Response scale:

1 = Strongly disagree

2 = Disagree

3 = Neither agree nor disagree

4 = Agree

5 = Strongly agree

Q1 - Artificial intelligence systems (software that can support or automate clinical or procedural decisions) have the potential to improve diagnosis, decision-making, or procedural performance in interventional pulmonology.

1

2

3

4

5

Q2 - The routine use of artificial intelligence during interventional pulmonology procedures may gradually reduce my ability to perform procedures independently without technological support (loss of procedural autonomy).

1

2

3

4

5

Q3 - I am familiar with the concept of automation bias, defined as the tendency to over-rely on artificial intelligence recommendations even when clinical judgment might suggest a different decision.

1

2

3

4

5

Q4 - Automation bias (excessive trust in artificial intelligence outputs without sufficient critical evaluation) may negatively affect patient safety and clinical decision-making in interventional pulmonology.

1

2

3

4

5

Q5 - The increasing use of artificial intelligence in clinical training may reduce opportunities for trainees to fully develop and consolidate procedural skills during their specialist education (upskilling inhibition).

1

2

3

4

5

Q6 - Training sessions performed without artificial intelligence or automated support are necessary to ensure the development and maintenance of independent clinical and procedural skills.

1

2

3

4

5

Q7 - Simulation-based training (using realistic models or virtual scenarios that replicate clinical procedures) is essential to maintain procedural competence in interventional pulmonology in the era of artificial intelligence.

1

2

3

4

5

Q8 - After the introduction of artificial intelligence in clinical practice, procedural performance should be systematically and longitudinally monitored to detect possible changes in operator competence over time.

1

2

3

4

5

Q9 - Current training programs in interventional pulmonology are not fully adequate to ensure safe integration of artificial intelligence while preserving independent procedural competence.

1

2

3

4

5

Q10 - Research focused on artificial intelligence–related loss of clinical and procedural skills (deskilling, meaning gradual reduction of competence due to reliance on technology) in interventional pulmonology should be considered a high scientific priority.

1

2

3

4

5

Q11 - Widespread reliance on artificial intelligence in interventional pulmonology may reduce institutional resilience in the event of system unavailability or malfunction (system fragility), potentially limiting the ability to safely perform procedures without technological support.

1

2

3

4

5

Q12 - Formal governance frameworks, including the definition of minimum non–AI-assisted procedural volume requirements, should be implemented to ensure the preservation of independent procedural competence and safe integration of artificial intelligence in interventional pulmonology practice.

1

2

3

4

5
