# Supplementary material for: Artificial Intelligence-Induced Deskilling in Interventional Pulmonology: An International Cross-Sectional Survey on Risk Perception and Mitigation Strategies
Source: Adv Respir Med. 2026 Jul 20;94(4):48. doi: 10.3390/arm94040048 (PMC13398125; doi:10.3390/arm94040048)
Supplement: Supplementary file 1 [file arm-94-00048-s001.zip › TABLE S1.pdf]

Item	Domain	n(1), %	n(2), %	n(3), %	n(4), %	n(5), %	Total	n(4+5), % (published)
**Q1**	Perceived clinical value of AI in IP	2 (2%)	4 (3%)	9 (8%)	57 (48%)	46 (39%)	118	103 (87%)
**Q2**	Risk of procedural deskilling with routine AI adoption	5 (4%)	9 (8%)	18 (15%)	47 (40%)	39 (33%)	118	86 (73%)
**Q3**	Prior familiarity with automation bias	10 (8%)	21 (18%)	42 (36%)	25 (21%)	20 (17%)	118	45 (38%)
**Q4**	Clinical relevance of automation bias (post-definition)	3 (3%)	6 (5%)	13 (11%)	53 (45%)	43 (36%)	118	96 (81%)
**Q5**	Upskilling inhibition due to AI over-reliance	3 (3%)	6 (5%)	11 (9%)	54 (46%)	44 (37%)	118	98 (83%)
**Q6**	Need for AI-free training sessions	3 (3%)	5 (4%)	11 (9%)	54 (46%)	45 (38%)	118	99 (84%)
**Q7**	Importance of simulation-based training	2 (2%)	5 (4%)	10 (8%)	56 (47%)	45 (38%)	118	101 (86%)
**Q8**	Importance of longitudinal monitoring	4 (3%)	7 (6%)	15 (13%)	51 (43%)	41 (35%)	118	92 (78%)
**Q9**	Inadequacy of current training programmes for AI integration	4 (3%)	8 (7%)	15 (13%)	50 (42%)	41 (35%)	118	91 (77%)
**Q10**	Deskilling as a research priority	2 (2%)	4 (3%)	7 (6%)	58 (49%)	47 (40%)	118	105 (89%)
**Q11**	Institutional fragility / system resilience risk	4 (3%)	9 (8%)	18 (15%)	48 (41%)	39 (33%)	118	87 (74%)
**Q12**	Support for governance frameworks (min procedural volume)	5 (4%)	10 (8%)	20 (17%)	46 (39%)	37 (31%)	118	83 (70%)

Table S1. Full distribution of Likert-scale responses (1 = strongly disagree to 5 = strongly agree) for each of the 12 survey items on AI-related deskilling in interventional pulmonology (n = 118 respondents). For each item, the number and percentage of respondents per response category are reported, together with the total and the combined agreement rate (n[4+5], %) already presented in the main manuscript.
